# Comparative Effect of ACTH and Related Peptides on Proliferation and Growth of Rat Adrenal Gland

**DOI:** 10.3389/fendo.2016.00039

**Published:** 2016-05-09

**Authors:** Claudimara Ferini Pacicco Lotfi, Pedro O. R. de Mendonca

**Affiliations:** ^1^Department of Anatomy, Institute of Biomedical Science, University of São Paulo, São Paulo, Brazil

**Keywords:** adrenal growth, ACTH, N-POMC, proliferation, cell cycle

## Abstract

Pro-opiomelanocortin (POMC) is a polypeptide precursor known to yield biologically active peptides related to a range of functions. These active peptides include the adrenocorticotropic hormone (ACTH), which is essential for maintenance of adrenal growth and steroidogenesis, and the alpha-melanocyte stimulation hormone, which plays a key role in energy homeostasis. However, the role of the highly conserved N-terminal region of POMC peptide fragments has begun to be unraveled only recently. Here, we review the cascade of events involved in regulation of proliferation and growth of murine adrenal cortex triggered by ACTH and other POMC-derived peptides. Key findings regarding signaling pathways and modulation of genes and proteins required for the regulation of adrenal growth are summarized. We have outlined the known mechanisms as well as future challenges for research on the regulation of adrenal proliferation and growth triggered by these peptides.

## Introduction

The primary function of the adrenal cortex is to produce steroids. Each zone of the adrenal cortex synthesizes different steroids in response to endocrine and paracrine stimuli. Adrenal function and maintenance of adrenal size are associated with regulation of adrenocortical growth, a topic that has been covered by other studies (1, 2). This review summarizes our understanding of growth regulators of the murine adrenal and highlights the action of adrenocorticotropic hormone (ACTH) and N-terminal peptides of pro-opiomelanocortin (N-POMC) in the control of proliferation and maintenance of the adrenal cortex.

## Pro-Opiomelanocortin in Murine

**Figure 1:**
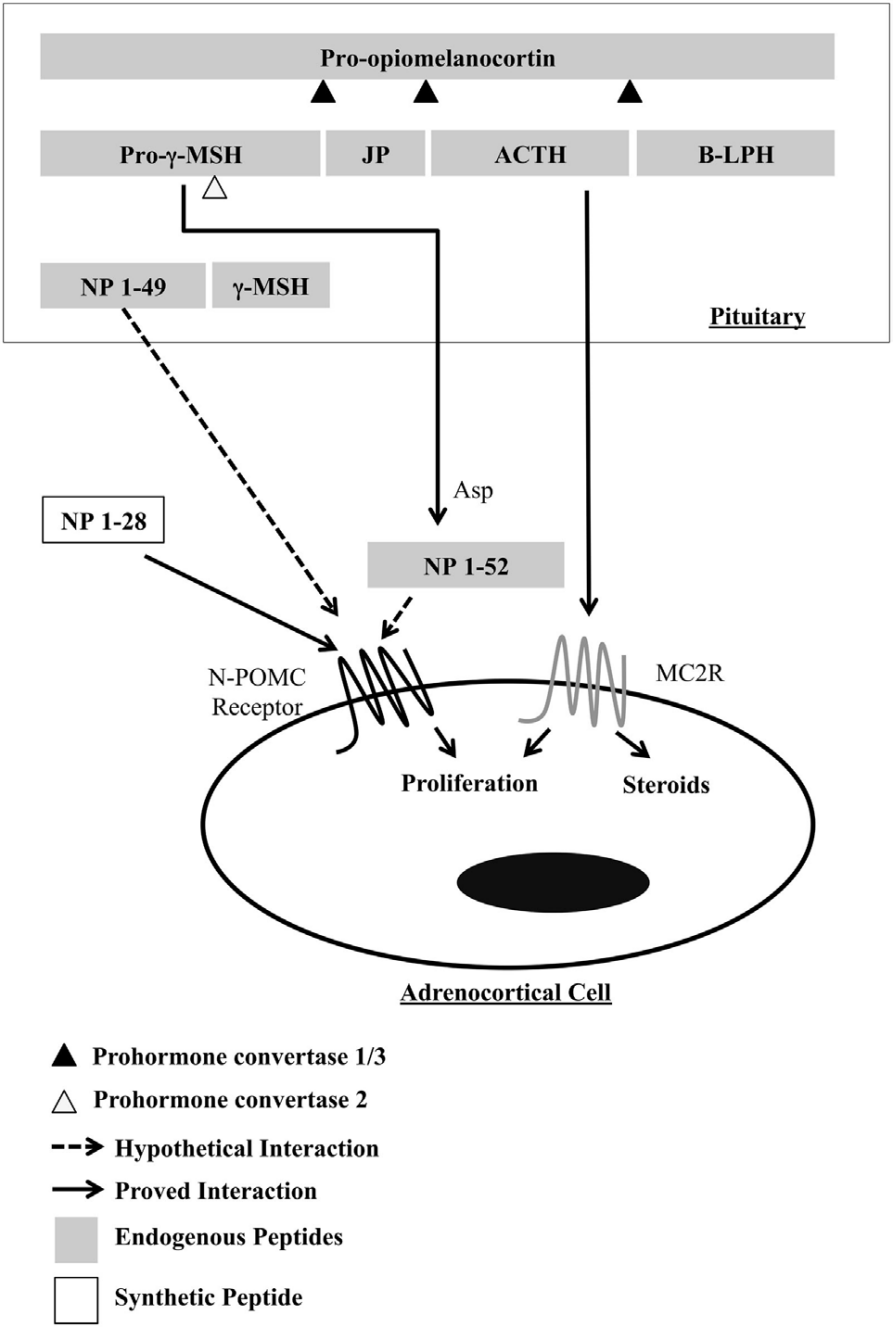
**Processing of POMC in the murine pituitary, highlighting the resulting peptides that act in the proliferation of adrenocortical cells**. γ-MSH, gamma-melanocyte-stimulating hormone; JP, joining peptide; ACTH, adrenocorticotropic hormone; B-LPH, beta-lipotropin; NP, N-terminal peptides of pro-opiomelanocortin; Asp, adrenal serine protease.

*Pomc* is a gene that belongs to the opioid/orphanin family. It is a highly conserved gene found from agnathan fish to mammals (3). In murines, this gene encodes a prohormone of 235 amino acids produced mainly by corticotropic cells in the pituitary gland. Post-translational processing at specific sites results in production of various smaller peptides, including peptide hormones with a range of physiological functions (Figure 1). In addition to the pituitary gland, POMC peptides are found in a diverse range of tissues, including the hypothalamus, skin, lung, gut, and pancreas (4). POMC transcripts found in these tissues are not full length, resulting in low levels of protein (5), and its function is not clear. POMC peptides in the circulation are derived mainly from the pituitary, and thus, the peptides produced in peripheral tissues act in an autocrine or paracrine way. The enzymes responsible for cleavage of POMC are called prohormone convertases (PC) and are of two types, PC1 and PC2. In the anterior lobe of the pituitary, the action of PC1 generates the main four POMC-derived peptides: the N-terminal peptide 1–74 (N-POMC 1–74 or pro-gamma-MSH), the joining peptide (JP), ACTH, and beta-lipotrophin (β-LPH). In the intermediate lobe, ACTH is cleaved by PC2 to produce alpha-melanocyte-stimulating hormone (α-MSH) and the corticotrophin-like intermediate peptide (CLIP); β-LPH is completely processed to γ-LPH and β-endorphin; and pro-gamma-MSH is cleaved to generate γ1- or γ3-MSH and N-POMC 1–49. γ1-MSH is found in humans but not in murines, as the cleavage site (a dibasic residue pair required for processing γ3-MSH into γ1-MSH) is missing in rodents (6). γ-MSH appears to potentiate the steroidogenic effect of ACTH in the adrenal gland, but the exact form of the peptide (γ1, γ2, or γ3) that produces this effect is still unclear (7). Currently, the concept of tissue-specific cleavage of POMC is acceptable, at least in the adrenal gland, where a serine-protease has been cloned and is responsible for cleaving the pro-gamma-MSH into a 52-residue peptide (8). The family of G-protein-coupled receptors named melanocortin receptors (composed of five members) is responsible for intermediating the action of POMC peptides.

## Proliferative Adrenal Cortex Responses to ACTH

The 39-amino acid peptide ACTH is the primary regulator of adrenal gland growth, maintenance, and function. Due to the actions of corticotropin-releasing hormone (CRH), arginine vasopressin, and other secretagogues, ACTH stimulates the pituitary corticotroph cells to release ACTH (9). ACTH binds to specific high-affinity receptors [melanocortin receptor 2 (MC2R)] located on the surface of adrenal cortical cells, stimulating the production of cortisol and corticosterone in murines, which in turn suppresses ACTH-releasing factors. ACTH increases rat adrenal weight by inducing both hyperplasia and hypertrophy in specific zones. In fact, administration of chronic exogenous ACTH in rats induces hyperplasia and hypertrophy in the outer and inner zona fasciculata, respectively (10), and results in a 70% increase of adrenal mass in rats (11). This phenomenon is also seen in knockout mice for both glucocorticoid and dopamine receptors showing elevated levels of circulating ACTH (12, 13). On the other hand, low levels of ACTH, such as those seen in animals submitted to hypophysectomy (14) or treated with dexamethasone (15), result in adrenal atrophy. When adrenal growth occurs to compensate for unilateral adrenalectomy in hypophysectomized rats, neither a decrease in circulating corticosterone nor elevated ACTH levels are observed (16), suggesting the action of neural mediation or other POMC-derived peptides.

## Molecular Mechanisms Implicated in ACTH Adrenocortical Growth

Abundant data relating to the signaling triggered by ACTH have been provided by experiments performed in cultured normal and tumoral adrenocortical cells. However, the *in vitro* action of ACTH on signaling pathways involved with adrenocortical growth is controversial and seems to depend on the cell type, the state of the responding cell, and other environmental signals from extracellular matrices (17, 18).

### ACTH *In Vitro*

In support of the mitogenic or antimitogenic action of ACTH, there are studies analyzing the regulation of ERK/MAPK and related pathways, in different cell types. In quiescent Y1 mouse adrenocortical tumor cells, the molecular mechanisms of cell cycle control comprise two contrasting control pathways for 1-nM ACTH treatment: (1) a mitogenic effect *via* induction of the *fos* and *jun* gene families and weak activation of ERK/MAPK; and (2) a cAMP/PKA-mediated antimitogenic mechanism comprising Akt pathway deactivation, cMyc degradation, and p27^Kip1^ induction (17, 19, 20). However, the ability of activate the ERK/MAPK was not interrupted in the cAMP-resistant mutant Y1 cells (Kin-8 cells) stimulated by ACTH, indicating the PKA not mediate the mitogenic action of ACTH (21). Arola and collaborators (22) also found an ACTH-inducible biphasic growth effect in rat adrenocortical cells in primary culture, in which a 7–70 nM ACTH-mitogenic effect transduced through the cAMP-mediated system and an ACTH-antimitogenic took place *via* a cAMP-independent pathway.

In another study performed with Y1 cells, the authors observed the inhibition of ERK/MAPK and c-Jun N-terminal kinases pathways through a PKC and Ca^2+^-dependent pathway (23), which favors an antimitogenic action of ACTH. In agreement, it was demonstrated by Bey and collaborators (24) that in Y1 cells, MAPK phosphatase-1 is a component of the ACTH signaling cascade, suggesting that ACTH can downregulate MAPKs. The antimitogenic and pro-apoptotic action of ACTH was reinforced in normal adrenal cells. In rat adrenocortical cells in primary culture, treatment for 3 days with 1-nM ACTH-induced apoptosis, activation of PKA/CREB but not ERK, and expression of c-Fos protein (25, 26). Also in support to antimitogenic action of ACTH, in bovine adrenocortical cells, angiotensin II activate MAPK after 5 min of treatment (EC50 = 0.1 nM), whereas ACTH does not stimulate ERK (27). Moreover, in rat adrenal zona glomerulosa cells, ERK activation blocked cell proliferation (28, 29).

In another widely used cellular model, the H295 human adrenocortical tumor cell line, which shares similarities with cells of the zona glomerulosa, ACTH stimulates ERK/MAPK signaling. However, in H295 cells, it has been described that MAPK stimulation by 100-nM ACTH depends on receptor internalization (30). On the other hand, in MC2R-transfected human embryonic kidney cells (31), 1-nM ACTH induces ERK phosphorylation that is partially PKA dependent. However, arrestin-coupled internalization does not involve any level of ACTH-dependent ERK phosphorylation (32). In summary, the analysis of signaling pathways involving the action of ACTH on different cell types and *in vitro* conditions gives support for an antimitogenic action of ACTH.

### ACTH *In Vivo*

In research involving depletion of the hypothalamic–pituitary–adrenal (HPA) axis using *in vivo* models with various approaches such as enucleation-induced adrenal regeneration (33), dexamethasone (Dex) treatment, and hypophysectomy, most but not all of the evidence converges on existing signals and pathways related to a mitogenic effect of ACTH.

Early response genes in the Fos and Jun gene families that form the transcriptional factor AP-1 and stimulate cellular proliferation (34) are induced by both ACTH and FGF2 infused in the rat adrenal gland *in situ* or in the adrenal cortex of hypophysectomized rats (35, 36). In enucleation-induced rat adrenal gland regeneration, the Fos gene was unregulated in the first 2 days of regeneration, while after 5 days of enucleation, downregulation of the Fos and Jun genes was observed (37).

Although it has been proposed that ACTH induces SAPK/JNK signaling activation and ERK/MAPK inhibition *in vivo* (23), results of chronic ACTH treatment in Dex-treated rats showed that ACTH is able to induce a sustained and progressive increase in ERK activation and proliferating cell nuclear antigen (PCNA) expression in all adrenal zones (38).

Other findings link the proliferative action of ACTH in Dex-treated rats with regulation of the cyclin-dependent kinase inhibitors (CDKIs) p27Kip1 and p57Kip2 in a time- and site-specific manner. A study shows that after Dex treatment, most of the cells expressed p27Kip1 but not p57Kip2. Subsequent ACTH treatment suppressed p27Kip1 expression and induced p57Kip2, while PCNA-expressing cells appeared mainly around the zona glomerulosa (39). Other cell-cycle regulators are also implicated in ACTH adrenocortical growth of Dex-treated rats. Besides increasing p27Kip1 expression, inhibition of the HPA axis downregulates cyclin D2 and D3 expression in the adrenal cortex. ACTH increases cyclin E and D3 expression, while it reduces expression of p27Kip1 protein in the outer and inner fraction preparations of adrenal cortex, respectively (40, 41). Moreover, the cell-cycle regulation is time dependent and zone specific. More recently, the Nek2 gene and its protein, together with the Notch gene, have also been shown to be involved in the cell cycle regulation triggered by ACTH (42).

The extracellular matrix (ECM) contributes to the regulation of cell proliferation and cell differentiation and therefore has a role in embryonic development and adult tissue homeostasis. Feige and colleagues have described the composition and expression of ECM components in the adult adrenal gland (43, 44). From the periphery to the center of the gland, the authors observed differential expression of fibronectin and laminin, which can be associated with specific activities of the cell components of the zones. However, studies conducted by the Gallo-Payet group show that ECM modulates basal and ACTH-induced cell functions, with fibronectin and collagen I and IV favoring steroid secretion, while laminin promotes proliferation (18). These findings illustrate the importance of the morphological changes associated with ACTH.

Despite the *in vivo* evidence that ACTH is the only factor that stimulates adrenal growth, other studies point in a different direction. As briefly described above, there are considerable data showing that ACTH inhibited growth of adrenal cells *in vitro*. In addition, Rao and colleagues (45) showed that rats treated with antiserum against ACTH had significant reduction of blood corticosteroids levels but did not exhibit adrenal atrophy. These and other results, which have been described in comprehensive reviews of the last 60 years of POMC research (46, 47), suggest that another factor distinct from ACTH has the ability to promote adrenal growth.

## Proliferative Adrenal Cortex Responses to N-POMC Peptides

In 1980, Estivariz and colleagues (48) extracted and purified pro-gamma-MSH from human pituitaries and showed that this peptide could not prevent adrenal atrophy in hypophysectomized rats. However, smaller N-POMC peptides (without the γ3-MSH portion) produced by trypsin digestion of pro-gamma-MSH or extracted from pituitary glands proved to be potent mitogens both *in vivo* and *in vitro*. In this section, we present information on the proliferative effect of the most important N-POMC peptides.

### N-POMC 1–28

N-POMC 1–28 was first isolated from human pituitary glands and later characterized as an extraction artifact (49). Even though N-POMC 1–28 is not an endogenous peptide, it has been extensively used to show the mitogenic effect of the N-POMCs. Moreover, the first 28 amino acids of the N-terminal portion of POMC have been shown to be essential to the triggering of adrenal cell proliferation. The mitogenic activity of N-POMC 1–28 has been demonstrated *in vivo* in murine models (40, 50, 51) and *in vitro* in Y1, NCI-H295R, and rat primary culture cells (26, 52, 53). Peripheral delivery of this peptide in *Pomc* KO mice does not promote any alterations in the adrenal gland (54) but may prevent atrophy of regenerating adrenal glands after hypophysectomy (55). These findings mean that besides promoting mitosis, this peptide may prevent apoptosis of adrenal cells. Indeed, our group has shown the anti-apoptotic effect of N-POMC 1–28 in adrenal glands of hypophysectomized rats (51). However, the molecular mechanisms underlying this effect are not clear. The positions of two disulfide bridges (between cysteine residues 2–24 and 8–20) seem to be essential to its biological activity (56).

### N-POMC 1–49

N-POMC 1–49 is an endogenous peptide produced and secreted by the intermediary lobe of the pituitary. It is one of the products from the cleavage of pro-gamma-MSH into smaller peptides. *In vitro* studies have shown that this peptide may promote proliferation of Y1 and NCI-H295R cells (52, 53). However, *in vivo* studies have shown that N-POMC 1–49 does not increase adrenal weight in fetal sheep when infused for 48 h (57). Interestingly, the presence of an O-linked glycan seems to be crucial for its proliferative effect. However, cleavage of pro-gamma-MSH in the pituitary occurs only if the O-linked glycan is not present in the molecule, resulting in an N-POMC 1–49 without the glycan [reviewed in Bicknell and Lowry (58)]. Clearly, the present data are controversial, and more assays must be done before it can be concluded that N-POMC 1–49 is the natural N-POMC peptide involved in adrenal proliferation and maintenance.

### Pro-Gamma-MSH

Pro-gamma-MSH is considered to be an active fragment found in the bloodstream at the same levels as ACTH (59). When infused into sheep fetus, this N-POMC peptide increased adrenal weight (57). However, as mentioned before, when administered in hypophysectomized rats, no effect on the adrenal weight was observed (60). Since the mitogenic peptides are located in the N-terminal portion of pro-gamma-MSH, a hypothesis of post-secretional cleavage occurring at the level of specific tissues has emerged. Indeed, Bicknell and collaborators (8) characterized a serine protease they named AsP (adrenal serine protease) that is present in the ECM of adrenal cells and is responsible for cleaving pro-gamma-MSH. The cleavage releases a peptide of 52 residues that induces proliferation of adrenal cortical cells. Moreover, Asp is capable of cleaving small basic substrates (e.g., arginine–arginine, lysine–arginine, etc.), generating N-POMC 1–49. These findings suggest the existence of an endogenous mitogenic N-POMC peptide, but no consensus about its identity has been reached.

## The Molecular Mechanism Involved in the N-POMC Proliferative Effect

The proliferative effect of N-POMC peptides has been established since the beginning of the 1980s, but its mechanism has begun to be unveiled only recently. The first study on this topic, conducted by Fassnacht and colleagues (52), concluded that N-POMC 1–28 promotes cell proliferation in NCI-H295R, Y1, and primary cultures of bovine adrenocortical cells by triggering a rapid activation of the ERK/MAPK but not the APK/JNK or p38 pathways. Pepper and Bicknell (53) corroborated those findings and showed that the upstream ERK regulators c-RAF and MEK were activated in Y1 and NCI-H295R cells treated with N-POMC 1–28 or N-POMC 1–49. In 2011, Mattos and collaborators (26) showed that ERK1/2 was activated in primary cultures of rat adrenocortical cells treated with N-POMC 1–28.

In 2014, we performed a PCR array to evaluate the effect of N-POMC 1–28 on the expression of key genes related to the control of the cell cycle. The genes *Nek2* and *Notch* were upregulated after treatment, suggesting that the proliferative effect of this peptide might be mediated by these genes (42). Additional research is needed to further elucidate the molecular mechanisms involved in the proliferative effect of N-POMC.

A fundamental question that has yet to be definitively answered is the identification of the receptor through which N-POMC peptides elicit their effects on adrenal growth. There have been two unsuccessful attempts to identify such a receptor (53, 58). In 2014, we joined efforts with Bicknell’s group and proposed a new approach to identifying this receptor. We cloned the most expressed orphan G-protein-coupled receptors in the rat adrenal gland and performed a magnetic cell separation assay using the N-POMC peptide attached to magnetic beads. A likely candidate for the N-POMC receptor was identified, confirmed by ligand-binding assays, and shown to be capable of activating the ERK pathway after stimulation with N-POMC. Further experiments are now been conducted to characterize *in vivo* and *in vitro* this potential adrenal N-POMC receptor. Final confirmation of the identity of the adrenal N-POMC receptor is essential for the understanding of cell proliferation in adrenocortical cells.

## Conclusion

In this paper, we summarize the current state of knowledge of the roles of ACTH and N-POMC in the proliferation of murine adrenal cells. We identify gaps in knowledge and describe conflicting results that need to be further investigated in order to fully understand the biology of this phenomenon. Examples of such urgently needed studies include gene array assays and pathway analysis to provide more data on the molecular mechanisms triggered by N-POMC peptides as well as to confirm the identity of the natural endogenous mitogenic N-POMC peptide. A holistic and interdisciplinary approach will be required, as none of these peptides or hormones act alone in nature. On the contrary, they trigger a net of responses and activate dozens of pathways simultaneously. When we begin to examine this phenomenon from a holistic perspective, we may come to truly understand the proliferative effect of these peptides.

## Author Contributions

The authors CL and PM contributed to the conception and design work.

## Conflict of Interest Statement

The authors declare that the research was conducted in the absence of any commercial or financial relationships that could be construed as a potential conflict of interest.
